# Diagnosis and Management of Aortic Valve Stenosis: The Role of Non-Invasive Imaging

**DOI:** 10.3390/jcm10163745

**Published:** 2021-08-23

**Authors:** Gloria Santangelo, Andrea Rossi, Filippo Toriello, Luigi Paolo Badano, David Messika Zeitoun, Pompilio Faggiano

**Affiliations:** 1 San Paolo Hospital, Division of Cardiology, Department of Health Sciences, University of Milan, 20142 Milan, Italy; gloriasantangelo@hotmail.it; 2 Division of Cardiology, Azienda Ospedaliero Universitaria Verona, 37126 Verona, Italy; andrea9rossi@gmail.com; 3 Fondazione IRCCS Ca’ Granda-Ospedale Maggiore Policlinico, Division of Cardiology, Department of Internal Medicine, University of Milan, 20122 Milan, Italy; filippo.toriello@gmail.com; 4 Department of Medicine and Surgery, University of Milano Bicocca, 20126 Milan, Italy; lpbadano@gmail.com; 5 Department of Cardiac, Metabolic and Neural Sciences, Istituto Auxologico Italiano, IRCCS, 20149 Milan, Italy; 6 Department of Cardiology, University of Ottawa Heart Institute, Ottawa, ON K1Y 4W7, Canada; DMessika-zeitoun@ottawaheart.ca; 7 Fondazione Poliambulanza, Cardiovascular Disease Unit, Via Leonida Bissolati, 57, 25100 Brescia, Italy

**Keywords:** aortic valve stenosis, echocardiography, classification, diagnostic imaging

## Abstract

Aortic stenosis is the most common heart valve disease necessitating surgical or percutaneous intervention. Imaging has a central role for the initial diagnostic work-up, the follow-up and the selection of the optimal timing and type of intervention. Referral for aortic valve replacement is currently driven by the severity and by the presence of aortic stenosis-related symptoms or signs of left ventricular systolic dysfunction. This review aims to provide an update of the imaging techniques and seeks to highlight a practical approach to help clinical decision making.

## 1. Introduction

Aortic valve stenosis (AS) is the most common heart valve lesion. The main etiologic forms of AS are rheumatic, degenerative and congenital. The common pathways of progressive valvular fibrosis and calcification lead to progressive thickening of the cusps with narrowing of the valve orifice and a left ventricular (LV) remodeling response [1].

Transthoracic echocardiography (TTE) remains the cornerstone of the severe AS definition, based on aortic valve area (AVA) < 1.0 cm^2^ or AVA indexed to body surface area—BSA—(AVAi) < 0.6 cm^2^/m^2^ and the trans-valvular pressure mean gradient (TPG) ≥ 40 mm Hg or peak aortic jet velocity (Vmax) ≥ 4 m/s [2]. Some patients with severe AS on the basis of AVA have a relatively low gradient despite a preserved left ventricular ejection fraction (LVEF) [3,4]. This situation raises uncertainty about the true severity of AS and the need for treatment [5]. Current guidelines suggest that the timing of aortic valve replacement (AVR) is dependent on the development of symptoms or reduction in LVEF [6,7]. Multimodality imaging techniques, such as computed tomography (CT), cardiovascular magnetic resonance (CMR) and positron emission tomography (PET), individualize management strategies in order to optimize the timing and choice of intervention.

The purpose of this review is to illustrate the imaging methods available today to assess the presence and severity of AS.

## 2. Echocardiographic Diagnosis and Pitfalls

TTE is a widely available, non-invasive and reliable technique which provides information on the severity of valve stenosis and its structural and functional impact on up- and down-stream cardiac structures.

### 2.1. Transvalvular Pressure Gradients

TPG is calculated starting from the velocity-time integral (VTI) of the envelope of the spectrum derived from the continuous Doppler from which the transvalvular antegrade velocity is estimated. It represents the maximum instantaneous gradient (that should not be confused with the so-called “peak-to-peak” gradient obtained during cardiac catheterization) obtained by measuring the peak pre- and post-stenotic Doppler velocities and applying the simplified Bernoulli equation. It was derived from the principle of conservation energy and is valid only for steady flows without viscous losses in one dimension. This modified version of the Bernoulli equation assumes a LV outflow tract (LVOT) velocity < 1 m/s and is valid only if the diameter of the sino-tubular junction is more than 30 mm [8]. While presenting, in fact, a linear correlation with catheter-measured values of TPG, it proved to be highly prone to errors in the presence of increased LVOT velocities and relevant pressure recovery (conversion of kinetic energy within the AS narrowing into pressure energy in the aortic root and the ascending aorta) resulting in overestimation. The effect of pressure recovery is particularly relevant in the case of low turbulence of the transvalvular flow and small size of the aortic root (justifying the difference between the TPG measured on the echocardiogram compared to that detected in the cath-lab) [9]. Furthermore, LVOT velocity cannot be assumed to be negligible in high output conditions such as aortic insufficiency, anemia, fever, thyrotoxicosis, arterio-venous fistula and Paget disease or when there is concomitant sub-valvular obstruction. In such cases, increased transvalvular flow can also be observed in patients with moderate AS. The consequence is that this method of calculating the TPG can be considered reliable only in case of severe stenosis and is inaccurate when AS is mild or moderate, even because the contribution of pressure recovery is more important in these cases. On the other hand, low flow conditions in which there is a reduction in LV SV and LV function can lead to an underestimation of the severity of AS by evaluating only Vmax and TPG. Hypertension contributes to the already increased afterload of AS and affects its evaluation because it may cause the underestimation of TPG [10]. In these cases, it is necessary to treat hypertension in order to reduce the double load that the LV faces during its ejection phase, defined as the valvulo-arterial impedance.

Finally, TPG is not able to represent the effective AVA in the presence of a variation of the LVEF in the same patient or following the positive inotropic stimulus during the dobutamine stress echocardiography (DSE), thus overestimating it. On the other hand, in patients with pseudo-stenosis, during DSE, the increase in SV will cause only a minimal increase in TPG and Vmax [11]. Besides, the measurement of Vmax and TPG can be subject to errors and lead to discordant results. However, even when the imaging quality is poor, they can successfully be determined in most patients. Accurate data recording requires multiple acoustic windows to determine the highest AS jet velocity and VTI. Apical (five-chamber view), suprasternal or right parasternal views most frequently yield the highest velocity [12]. The use of multiple views limits the risk of underestimation by 20%. Another important source of underestimation is the suboptimal alignment of Doppler recordings with the aortic jet. Overestimation, instead, occurs in the case of the simultaneous presence of mitral regurgitation (MR), when dynamic intraventricular obstruction velocities are interpreted as aortic jets or when a beat following a long diastole is included in measurements [13].

**Figure 1:**
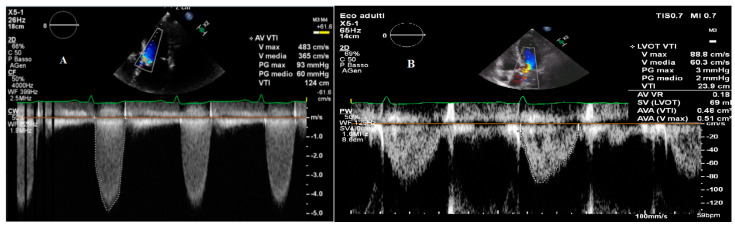
(**A**) Recording of the peak velocity through a stenotic aortic valve in the apical five-chamber view by continuous-wave Doppler. (**B**) Recording of the peak velocity through Left ventricular outflow tract in the apical three-chamber view by pulse-wave Doppler. The images were executed with Philips EPIQ 7 (Philips Medical System, Bothell, WA, USA).

Figure 1A shows a recording of Vmax through a stenotic aortic valve in the apical five-chamber view by a continuous-wave Doppler.

### 2.2. Aortic Valve Area

The effective orifice AVA is assessed by the continuity equation which assumes that the SV at the valve orifice level is equal to that at the LVOT. It requires the measurement of three parameters: the LVOT cross-sectional area (normally determined as π × (LVOT diameter/2)^2^), the LVOT VTI (determined by pulsed wave Doppler) and the transvalvular VTI determined by continuous wave Doppler. From these, the AVA can be calculated as the product of the LVOT cross-sectional area and LVOT VTI divided by the continuous wave Doppler aortic flow VTI (Figure 1B).

**Figure 2:**
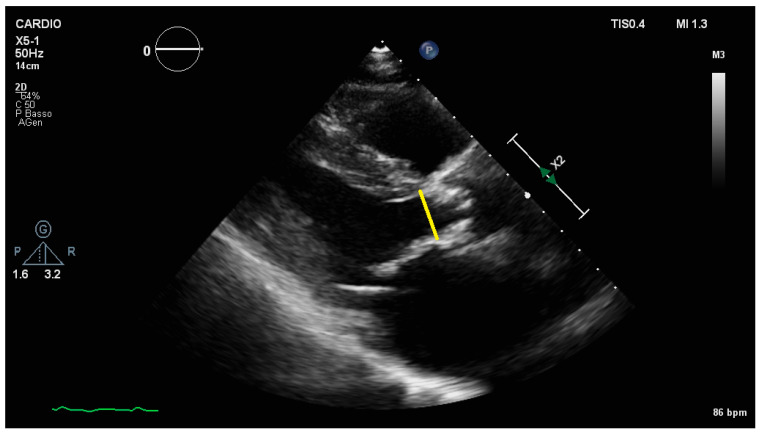
Measurement of the diameter of the Left ventricular outflow (yellow line) from the parasternal long-axis view, during systole, directly below the base of the aortic leaflets, and from the internal surfaces of the interventricular septum and the posterior aortic wall.

The main limitation in the assessment of AVA is the estimate of the LVOT diameter. It should be measured (and repeated at least five times) in the parasternal long-axis view, using the zoom mode, in mid systole. European and American society guidelines recommend its estimate at the aortic annulus level (i.e., at the level of leaflet insertion) rather than more apically (5–10 mm below the annulus), where the cross-sectional shape is more elliptical, more irregular (because of the frequent septal bulge) and more dynamic (greater changes between diastole and systole) than at the level of the aortic annulus [14], in order to provide higher reproducibility and to measure diameter and pulse Doppler at the same anatomical level (Figure 2) [6,7]. The main difficulties lie in the fact that it is technically challenging to measure, especially in elderly patients with calcific AS, poor echogenicity windows or in the presence of mitral valve prostheses; it is squared in the continuity equation and so a 1 mm difference can cause 10% variation in LV SV. In addition, all the sources of error already mentioned and linked to the calculation of the TPG, which apply in the same way to the estimate of the transvalvular VTI, must be considered and will result in an overestimation of the residual aortic valve orifice. Potential assessment problems related to incorrect positioning of the pulse wave Doppler in the calculation of the LVOT VTI are often not taken into consideration. A positioning of the sample volume too far from the aortic valve plane leads to an overestimation of the severity of the AS. Conversely, if positioned too close, within the flow acceleration, it will underestimate AS severity. Leye et al. have shown that LVOT diameter, estimated by TTE and transesophageal echocardiography (TEE) measurements, was significantly associated to BSA and LVOT diameter, derived from a linear regression linked to BSA independently of gender, and provided an acceptable approximation of the AVA. The present equation may be used as a safeguard when LVOT diameter measurement is difficult or not possible with TTE [15].

In patients with small body size, it may be helpful to use AVAi to avoid overestimation of stenosis severity. The role of indexing for body size is controversial, because the current algorithms for defining body size do not necessarily reflect the normal AVA in obese patients and because AVA does not increase with excess body weight [7]. Recently, Vulesevic et al. have shown that severe AS should be defined as an AVA < 1 cm^2^ or an AVA index to height < 0.6 cm^2^/m rather than an AVAi value of 0.6 cm^2^/m [16]. An alternative is represented by the direct planimetry of the valve orifice by 3D TEE which is useful for grading AS severity in patients with a poor transthoracic acoustic window. The measurement of effective AVA is possible using the continuity equation provided that a good Doppler alignment to the aortic valve jet is achieved from the trans-gastric view [17] TEE is also more optimal for annular sizing, the assessment of coronary obstruction, positioning and paravalvular leak [18]. Finally, the ratio between the sub-valvular and the Vmax is a simplified version of the continuity equation that ignores the LVOT diameter and thus is not subject to errors related to its measurement. A value < 0.25 is indicative of severe AS.

The AS severity classification is shown in Table 1.

### 2.3. Exercise Testing

Semi-supine bicycle exercise on the tilted bed allows for continuous 2D and Doppler echocardiographic evaluation of the valve, ventricle and its hemodynamic consequences during exercise [19]. The initial workload of 25 W for 2 min is usually proposed, and it is increased every 2 min by 25 W. The total exercise time, maximum workload, reason for stopping the test, peak heart rate and blood pressure are recorded. Complete resting echocardiography is performed at rest, prior to exercise, with the aim of evaluating the severity of AS and its consequences (extravalvular cardiac damage or stage of AS). The evaluation of LV diastolic parameters, MR and the presence of ischemia are part of the stress echocardiography protocol, although their prognostic values have not been evaluated so far [20]. Exertional dyspnea could also be explained by diastolic dysfunction and an increase in LV filling pressure (estimated by E/e’) and an increase in MR severity with exercise, whereas ischemia could also be the cause of chest pain in patients with AS. Therefore, due to the lack of large-scale prospective randomized studies, none of these echocardiographic parameters currently represent an indication for intervention in asymptomatic patients with severe AS. Exercise echocardiography could help identify a subset of patients with early and subtle but harmful consequences of AS, who may require AVR earlier and might benefit from a more frequent follow-up. The inclusion in the rest protocol of LV global longitudinal strain (GLS) and valvulo-arterial impedance (defined as systolic arterial pressure + mean aortic valve gradient/LV stroke volume index) has been shown to have an incremental prognostic value. In a recent study on 504 patients with asymptomatic severe AS and preserved LVEF, a LV-GLS value < −17% and valvulo-arterial impedance >4.5 mm Hg/mL/m^2^ were associated with an increased risk of death at 5 years [21]. It should be considered in asymptomatic patients with severe AS after a careful history, in order to unmask symptoms or abnormal blood pressure responses [6]. Although a negative exercise test result is a reassuring finding in younger patients, the predictive value of the test is lower in older adults and may be further improved when combined with an echocardiographic assessment of LV function, TPG and pulmonary arterial pressure [22]. There are a few studies on stress echocardiography in patients with moderate AS, suggesting that these patients could represent a higher risk group for disease progression and should be followed more closely (every 6–12 months) [23].

### 2.4. Strain

Reduced LV GLS is an early marker of impaired contractile function when LVEF is still preserved and is associated with the presence of myocardial fibrosis [24]. Small series in asymptomatic patients have also linked GLS with subsequent cardiac events [25,26]

Anyway, GLS clinical use and its adoption into guidelines as a formal indication for treatment is limited by a lack of standardization between vendors and an overlap in values amongst those with health and disease [27]. Furthermore, the analysis of left atrial strain during the atrial reservoir phase shows that a value lower than 21% was associated with a 2.88-fold increased risk for mortality or hospitalization [28] after adjustment for age, NYHA class and presence of coronary artery disease (CAD).

### 2.5. Extra-Valvular Cardiac Damage

The importance of anatomical or functional cardiac consequences of AS, with the exception of a LVEF < 50%, is not taken into account in the AVR decision algorithm, but in 2017, a new staging classification of AS was proposed in symptomatic subjects [29]. It was based on the absence or presence of cardiac damage as follows: no extra-valvular cardiac damage (stage 0), LV damage (stage 1), LA or mitral valve damage (stage 2), pulmonary vasculature or tricuspid valve damage (stage 3) or right ventricular damage (stage 4). Tastet et al. [30] have demonstrated that stage classification is associated with a 30% increase in risk of mortality per stage of disease in asymptomatic AS. These authors confirmed the lack of sensitivity of symptoms to identify the presence and extent of cardiac damage and emphasized the importance of using a cardiac damage staging approach, based on Doppler echocardiographic parameters, to guide the therapeutic management in patients with asymptomatic moderate to severe AS.

## 3. Current Guidelines

The recent American guidelines [7] recognize different stages of AS, ranging from patients at risk of AS (Stage A) or with progressive hemodynamic obstruction (Stage B) to severe asymptomatic (Stage C) and symptomatic AS (Stage D). Each stage is defined by patient symptoms, valve anatomy, valve hemodynamics and changes in the LV and vasculature. Severe symptomatic AS with low flow-low gradient (LF-LG) is designated D2 (with a low LVEF) or D3 (with a normal LVEF).

The European guidelines [6], based on the AVA and TPG, define four categories of AS when AVA < 1 cm^2^:

High-gradient severe aortic stenosis (AVA < 1 cm^2^, TPG > 40 mmHg): if high flow status (i.e., anemia, hyperthyroidism, arteriovenous shunt) is excluded the stenosis is severe.

LF-LG AS with reduced LVEF or “Classical”: (AVA ≤ 1 cm^2^, a TPG < 40 mmHg, SV index ≤ 35 mL/m^2^ and LVEF < 50%): the decreased LVEF is generally due to high afterload from valvular disease and intrinsic myocardial impairment (i.e., ischemic heart disease, diffuse/focal myocardial fibrosis secondary to AS/hypertension or concomitant cardiomyopathies) [31]. Low dose DSE and measurement of the degree of aortic valve calcification (AVC) using CT distinguish truly AS from pseudo-severe AS,

LF-LG AS with preserved LVEF or “Paradoxical” (AVA ≤ 1 cm^2^, TPG < 40 mmHg, SV index ≤ 35 mL/m^2^ and LVEF ≥ 50%): due to the fact that TPG is more dependent on transvalvular volumetric flow rate (FR) (intended as the volume of fluid which passes per unit time and calculated as the SV divided by the LV ejection time) than on SV index, some investigators propose to define low-flow as a mean FR < 200 mL/s [32]. These patients are more frequently women, with pronounced concentric remodeling and small LV cavity, diastolic dysfunction and reduced LV systolic longitudinal function despite the preserved LVEF [33]. The degree of AVC, obtained from evaluation by CT, can be used to identify severe AS [6].

Normal-Flow Low-Gradient AS with preserved LVEF (AVA ≤ 1 cm^2^, TPG < 40 mmHg, SV index > 35 mL/m^2^ and LVEF ≥ 50%): European guidelines advocate the idea that these patients have only moderate AS, but a significant proportion have truly severe AS and would benefit from AVR if symptomatic [34]. This pathophysiological entity is explained by factors such as the presence of an actually decreased FR despite a normal SV index: bradycardia, systemic hypertension and/or reduced arterial compliance have been shown to decrease the SV index, prolong LV ejection time and cause a drop in mean transvalvular FR and/or TPG [35].

The two different classification systems for the severity of AS, according to European and American guidelines, are summarized in Table 2.

Furthermore, the American guidelines make no mention of biomarkers, whereas the European guidelines currently indicate that AVR is reasonable (Class IIa) in an asymptomatic patient with a “markedly elevated natriuretic peptide level” defined as levels three-fold greater than the age- and sex-corrected normal range, confirmed by repeated measurements without other explanations [6].

## 4. Multimodality Imaging for Discordant (Low-Gradient) AS

A significant proportion of patients with severe AS presents with discordant grading (i.e., AVA ≤ 1 cm^2^ and a TPG < 40 mmHg) [4]. The first step is to confirm the validity of echocardiographic measures of AS severity and to treat hypertension. The next step is to differentiate severe from non-severe AS with the use of low dose DSE, in patients with classical LF-LG AS, while AVC measured by CT is preferred in patients with paradoxical LF-LG AS, as well as in those with inconclusive results with DSE. CMR and PET could allow a detailed assessment of the AS valve and the myocardial remodeling response.

### 4.1. Dobutamine Stress Echocardiographic

The dobutamine infusion protocol consists of 5 min increments of 5 μg/kg/min up to a maximum dosage of 20 μg/kg/min. In response to inotropic stimuli, severe AS is characterized by an increase in Vmax with values ≥ 4 m/s (TPG ≥ 40 mmHg) at any FR, but with AVA remaining ≤ 1.0 cm^2^. In contrast, pseudo-severe AS is identified by an increase in AVA > 1.0 cm^2^ with flow normalization and TPG reduction. In addition, the absence of flow reserve (also termed contractile reserve; increase in SV < 20%) has prognostic implications because surgical aortic valve replacement (SAVR) in this setting comes with a weaker recommendation (IIb, level of evidence C) [6], but prognosis in such patients is dismal with medical management alone, and as such transcatheter aortic valve implantation (TAVI) may be a more favorable option [27].

**Figure 3:**
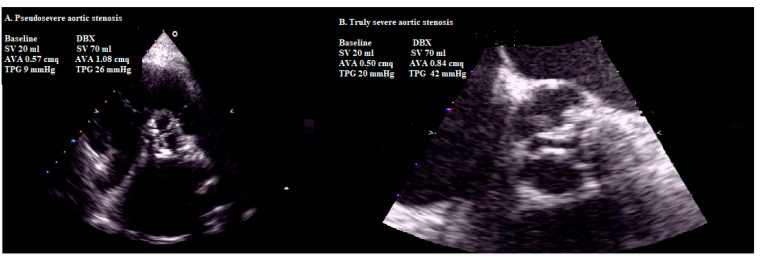
The figure shows the case of valve with pseudo-severe aortic stenosis (**A**) at low (rest) and normal flow rate (dobutamine) and that of valve with truly severe aortic stenosis (**B**) at low and normal flow rate. SV: stroke volume; AVA: aortic valve area; TPG: transvalvular pressure gradient. DBX: Dobutamine. See the text for details. A projected AVA ≤ 1 cm^2^ is considered severe. In many ways, the concept of projected AVA outperforms the traditional flow-reserve concept, which may be intrinsically flawed due to the complex interaction between decreased contractility, increased afterload and altered geometry in classical LF-LG AS [38]. However, many patients with paradoxical LF-LG AS have small ventricles, with concentric remodeling and a high prevalence of atrial fibrillation and isolated upper septal hypertrophy, which may lead to serious side effects such as LVOT dynamic obstruction with hypotension and syncope; thus, DSE should be stopped as soon as increase in flow is sufficient.

However, a relevant proportion of patients do not have flow reserve and/or have inconclusive results at DSE. The changes in TPG and AVA during DSE largely depend on the magnitude of flow augmentation achieved, which may vary considerably from one patient to another (Figure 3). Therefore, this approach may be misleading [36]. Hence, the concept of projected AVA (i.e., the projected AVA at a normal FR of 250 mL/s induced by dobutamine) was developed [36,37] After an increase of ≥15% in FR, using AVA and FR [Q], projected AVA is calculated as follows:

(1) $$AVA_{Proj}=AVA_{Rest}+\frac{AVA_{Peak}-AVA_{Rest}}{Q_{Peak}-Q_{Rest}}\times (250-Q_{Rest)}$$

### 4.2. Computed Tomography in AS

AVC by CT is a load independent, low radiation (<1 mSv), quantitative and extremely reproducible technique to assess aortic valve thickening, which requires an ECG-gated non-contrast acquisition [6]. Quantification relies on the Agatston method using semi-automated software and provides a flow-independent quantitative assessment of AS anatomical severity. Due to sex differences in AS pathophysiology (women present with less calcification than men), specific cut-offs defining severe AS differ for women (≥1200 Agatston Units—AU) and men (≥2000 AU) [39]. Patients with particularly large or small aortic annuli would likely benefit from the AVC indexation to BSA, defined as AVC density [40]. AVC has been shown to be a powerful independent predictor of hemodynamic progression in AS, which may further individualize optimal timing of follow-up and/or intervention [41]. However, it should be emphasized that non-contrast CT only captures mineralized tissues and would underestimate AS severity in younger patients with a bicuspid aortic valve in which the stenosis is predominantly caused by non-calcified tissues [42].

**Figure 4:**
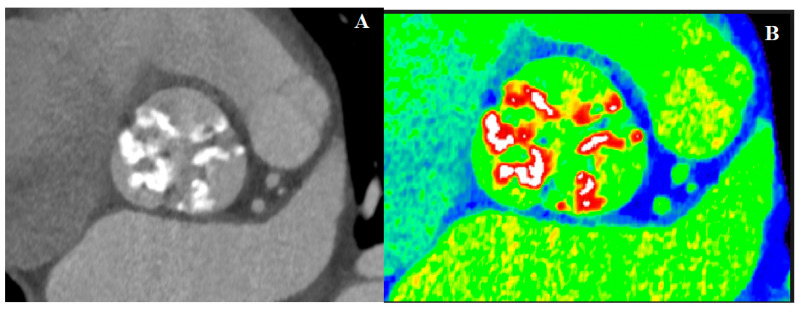
Measurement of aortic valve calcifications on representative images. (**A**) Low dose chest CT scan image focused on the aortic valve of an 86-year-old woman with low-flow low-gradient aortic stenosis and 9000 Agatston score. (**B**) In white color number in Hounsfield Units (HU) to quantify aortic valvular calcium on contrast-enhanced scans. Here, equal to 1000 HU. Courtesy of Marianna Adamo.

In Figure 4, we report an example of AS severity assessment by CT-AVC.

### 4.3. Cardiovascular Magnetic Resonance

CMR emerged as an alternative and non-invasive method which avoids many of the pitfalls of other imaging techniques: sedation/anesthesia for TEE and contrast exposure of CT [43]. Moreover, CMR can offer a detailed identification and quantification of myocardial fibrosis, which has been implicated in prognosis post-SAVR or TAVI [44,45] and often predates a reduction in LVEF [46,47]. Late gadolinium enhancement (LGE) correlates with the degree of interstitial fibrosis on an endomyocardial biopsy [48]. Treibel et al. have recently demonstrated that myocardial fibrosis in AS comprises both diffuse reactive interstitial (reversible) and more focal replacement (irreversible) forms, often with a subendocardial-to-epicardial gradient [49]. In case of LF-LG, severe AS with normal or reduced LVEF, CMR has currently a limited role in terms of diagnosis and is not included in recent guidelines. Due to the low-flow condition, the maximum potential AVA may be underestimated by CMR direct planimetry of the stenotic valve. CMR is, therefore, not helpful in differentiating between pseudo-severe and severe AS [50].

Strain imaging on CMR can evaluate and quantify myocardial deformation appearing before any identifiable changes in LVEF, especially in patients with suboptimal echocardiography image quality [51].

**Figure 5:**
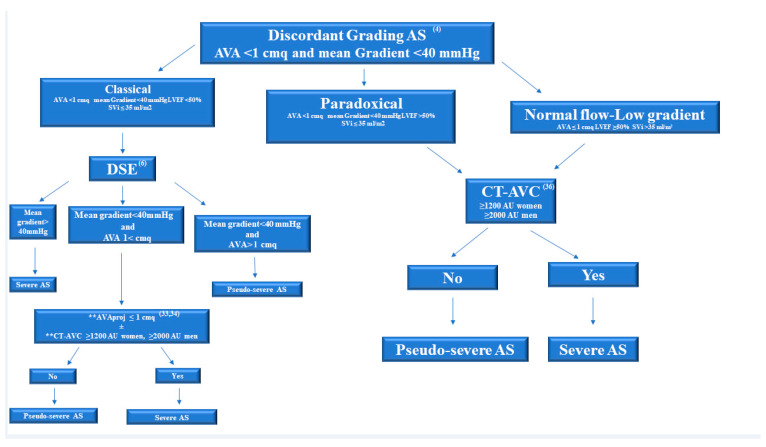
Stepwise integrated approach for the assessment of discordant grading aortic stenosis. AS: aortic stenosis; AVA: Aortic valve area; LVEF: Left ventricular ejection fraction; SVi: stroke volume index to body surface area; DSE: dobutamine stress echocardiography; AVA proj: projected area at standardized normal flow rate of 250 mL/s. AVA proj ≤ 1 cm^2^ is considered severe; CT-AVC: computed tomography aortic valve calcium scoring (severity cut-offs: women ≥ 1200 AU and men ≥ 2000 AU); ** AVA proj and CT-AVC are used because the stenosis severity often remains indeterminate at the outset of DSE, or echocardiographic assessments are discordant.

Figure 5 shows a stepwise integrated approach for the assessment of low gradient AS.

### 4.4. Positron Emission Tomography

PET-CT offers novel insights into the complex pathophysiological processes driving AS, including inflammation, calcium deposition and ossification [52]. Radiolabeled sodium fluoride (^18^F-NaF) has an affinity for microcalcification. It may also localize to areas of valve degeneration and areas predisposed to progressive degeneration. This technique offers considerable promise as a biomarker of disease activity, as a means of predicting disease progression [53], and it may also identify therapeutic targets for novel pharmacotherapy [54].

## 5. Indications for Intervention

Current recommendations [6,7] for AVR in AS patients rely solely on mean TPG, AVA and the presence of symptoms. It is also necessary to take in account the Society of Thoracic Surgeons score or Euroscore II, frailty and the compromise of other major organ systems [6]. Management of asymptomatic severe AS remains controversial. Predictors of symptom development and adverse outcomes in these patients include clinical characteristics (e.g., older age, presence of atherosclerotic risk factors), echocardiographic parameters (rate of hemodynamic progression, increase in TPG > 20 mmHg with exercise, pulmonary hypertension) and biomarkers [6]. Nevertheless, new insights into the pathophysiology of AS patients, advances in diagnostic imaging and the evolution of TAVI are fueling interest in the management of asymptomatic patients with severe AS. A less invasive intervention than the SAVR could plausibly justify preventive AVR in these patient subgroups rather than waiting for the appearance of the first symptoms [55]. The choice between SAVR vs. TAVI should be made by a multidisciplinary Heart Team, taking into account the clinical characteristics of the patient, the association with other valve disease, CAD, comorbidities and frailty.

Finally, the use of balloon aortic valvuloplasty in calcified AS is generally limited to severely symptomatic patients as a bridge to SAVR/TAVI or requiring an urgent non-cardiac surgery [6].

**Figure 6:**
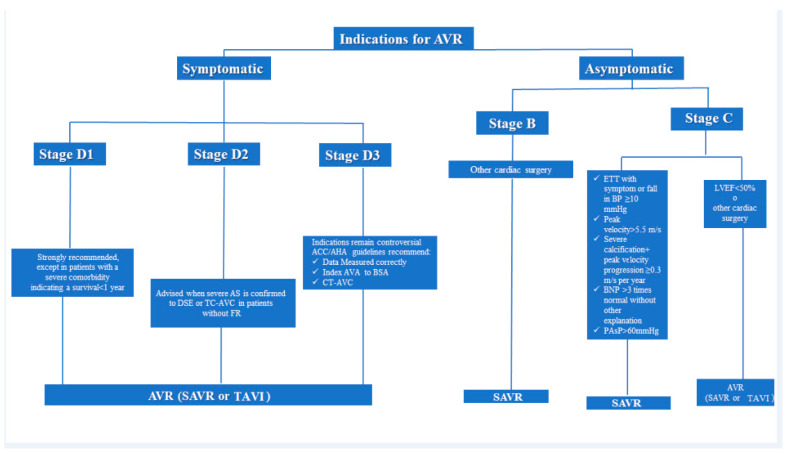
Current recommendation from European and American guidelines for aortic valve replacement in aortic stenosis. AVR: Aortic Valve Replacement. AS: Aortic Stenosis. DSE: Dobutamine Stress Echocardiography. SAVR: Surgical aortic valve replacement. TAVI: Transcatheter aortic valve replacement. ACC/AHA: American College of Cardiology/American Heart Association. AVA: Aortic valve area. BSA: Body Surface Area. TC-AVC: computed tomography aortic valve calcium scoring. LVEF: left ventricular ejection fraction. PAsP: Pulmonary artery systolic pressure. ETT: exercise treadmill test BP: blood pressure.

Current recommendations for AVR are summarized in Figure 6.

### Special Patient Populations

In patients requiring coronary artery bypass graft surgery (CABG), if AS is severe or the patient has symptoms, AVR should be performed in conjunction with CABG. Most operators agree that coronary revascularization, in patients referred to TAVI, should be performed before AVR when CAD involves the proximal segments of major epicardial coronary arteries, in the presence of significant renal dysfunction and if there is no concern about prolonged dual antiplatelet therapy [56].

Severe AS frequently coexists with severe MR, but the absence of clinical trials suggests careful evaluation to distinguish patients who might benefit from a double valve intervention [6].

Up to one third of patients with paradoxical AS might have concomitant cardiac amyloidosis (CA), commonly due to wild-type transthyretin [57]. Compared to patients with lone AS, those with AS and CA are older, have worse functional status, higher circulating N-terminal pro-brain natriuretic peptide and troponin levels [58]. Some studies suggest that amyloid substance may deposit within the aortic valve leaflets and thus lead to the development of AS despite absent or minimal leaflet calcification. In such cases, non-contrast CT, which captures only the calcified component of the valve leaflet tissue, would underestimate the severity of this amyloid-related AS [59].

Typical LGE patterns, and elevated T1 values and extracellular volume fraction as a result of amyloid infiltration [60], may elevate the index of suspicion of AS e CA, although the use of bone scintigraphy is supported by recent expert consensus recommendations to confirm the diagnosis non-invasively [61]. TAVI has been shown to improve outcomes in CA and SA with no increased periprocedural complications and mortality compared with AS alone [62].

## 6. Conclusions

AS is the most common type of valvular heart disease. The therapeutic decision essentially depends on symptomatic status and stenosis severity, but the extent of cardiac damage associated has important prognostic implications after AVR. Echocardiography is the gold standard for the diagnosis of AS. However, multimodality imaging has an important role in the management of AS in order to find out subclinical pathophysiological changes, improve a patient’s selection candidate to SAVR or TAVI, stratify risk and improve procedural outcomes.

## Figures and Tables

**Table 1:** Recommendations for grading of AS severity.

Parameters	Mild AS	Moderate	Severe
Vmax (m/s)	2.6–2.9	3.0–4.0	≥4.0
Mean gradient (mmHg)	<20	20–40	≥40
AVA (cm^2^)	>1.5	1.0–1.5	<1
AVAi (cm^2^ /m^2^)	>0.85	0.6–0.85	<0.6

AS: aortic stenosis; Vmax: Peak Aortic Jet Velocity; AVA: Aortic Valve Area; AVAi: Aortic Valve Area index to Body Surface Area.

**Table 2:** Comparison between the different classification systems of the severity of AS in the European and American guidelines.

2017 ESC/EACTS Guidelines for the Management of Valvular Heart Disease					
High-Gradient AS		Low-Flow, Low-Gradient AS with Reduced LVEF	Low-Flow, Low-Gradient AS with Preserved LVEF		Normal-Flow, Low-Gradient AS with Preserved LVEF
AVA < 1 cm^2^, ΔP > 40 mmHg		AVA < 1 cm^2^, ΔP < 40 mmHg, LVEF < 50%, SVi ≤ 35 mL/m^2^	AVA < 1 cm^2^, ΔP < 40 mmHg, LVEF ≥ 50%, SVi ≤ 35 mL/m^2^		
**2020 ACC/AHA Guideline for the Management of Patients with Valvular Heart Disease**					
**Stage**	**Definition**	**Valve Anatomy**	**Valve Hemodynamics**	**Hemodynamic Consequences**	**Symptoms**
A	At risk of AS	BAV or Aortic sclerosis	AV_max_ < 2 m/s	None	None
B	Progressive AS	Mild/moderate leaflet calcification or rheumatic valve changes	Mild AS: AV_max_ 2.0–2.9 m/s or mean Δ < 20 mmHg Moderate AS: AV_max_ 3.0–3.9 m/s or Δ 20–39 mmHg	Early left ventricular diastolic dysfunction may be present Normal LVEF	None
C1	Asymptomatic severe AS	Severe leaflet calcification/fibrosis or congenital stenosis with severely reduced leaflet opening	AV_max_ ≥ 4 m/s or Δ ≥ 40 mmHg AVA typically is ≤1.0 cm^2^, but not required to define severe AS Very severe AS is an AV_max_ ≥ 5 m/s or Δ ≥ 60 mm Hg	Left ventricular diastolic dysfunction Mild left ventricular hypertrophy Normal LVEF	None
C2	Asymptomatic severe AS with reduced LVEF	Severe leaflet calcification/fibrosis or congenital stenosis with severely reduced leaflet opening	AV_max_ ≥ 4 m/s or Δ ≥ 40 mmHg AVA typically is ≤1.0 cm^2^, but not required to define severe AS	LVEF < 50%	None
D1	Symptomatic severe high- gradient AS	Severe leaflet calcification/fibrosis or congenital stenosis with severely reduced leaflet opening	AV_max_ ≥ 4 m/s or Δ ≥ 40 mmHg AVA typically is ≤1.0 cm^2^, but not required to define severe AS	Left ventricular diastolic dysfunction Left ventricular hypertrophy Pulmonary hypertension may be present	Exertional dyspnea, angina or pre-syncope or syncope, decreased exercise tolerance or HF
D2	Symptomatic severe low-flow, low-gradient AS with reduced LVEF	Severe leaflet calcification/fibrosis with severely reduced leaflet motion	AVA ≤ 1.0 cm^2^ with resting AV_max_ < 4 m/s or ΔP < 40 mmHg Dobutamine stress echo shows AVA < 1.0 cm^2^ with AV_max_ ≥ 4 m/s at any flow rate	Left ventricular diastolic dysfunction Left ventricular hypertrophy LVEF < 50%	HF Angina Syncope or pre-syncope
D3	Symptomatic severe low-gradient AS with normal LVEF or paradoxical low-flow severe AS	Severe leaflet calcification/fibrosis with severely reduced leaflet motion	AVA ≤ 1.0 cm^2^ with resting AV_max_ < 4 m/s or Δ < 40 mmHg AND SVi ≤ 35 mL/m^2^ measured when patient is normotensive (systolic blood pressure <140 mmHg)	Increased left ventricular relative wall thickness Small left ventricular chamber with low SV Restrictive diastolic filling Normal LVEF	HF Angina Syncope or pre-syncope

AS: Aortic valve Stenosis; AVA: Aortic Valve Area; Δ: mean pressure gradient; LVEF: Left Ventricular Ejection Fraction; SVi: Stroke Volume index; MSCT: Multislice Computed Tomography; BAV: Bicuspid Aortic Valve; AV_max_: Aortic maximum velocity; HF: Heart Failure; ESC/EACTS: European Society of Cardiology/European Association for Cardio-Thoracic Surgery. ACC/AHA: American College of Cardiology/American Heart Association.

## Data Availability

Not applicable.

## Abbreviations

AS	Aortic stenosis
AU	Agatston Units
AVA	Aortic Valve Area
AVAi	Aortic Valve Area index to Body Surface Area
AVC	Aortic valve calcium scoring
AVR	Aortic Valve Replacement
BSA	Body Surface Area
CA	Cardiac amyloidosis
CABG	Coronary artery bypass graft surgery
CAD	Coronary Artery Disease
CMR	Cardiovascular magnetic resonance
CT	Computed tomography
DSE	Dobutamine stress echocardiography
18F-NaF	Radiolabeled sodium fluoride
FR	Flow rate
GLS	Global Longitudinal Strain
LFLG	Low Flow-Low Gradient
LGE	Late gadolinium enhancement
LV	Left ventricular
LVEF	Left ventricular ejection fraction
LVOT	Left ventricular outflow tract
MR	Mitral Regurgitation
PET	Positron emission tomography
SAVR	Surgical aortic valve replacement
SV	Stroke Volume
TAVI	Transcatheter Aortic Valve Implantation
TEE	Transesophageal echocardiography
TPG	Transvalvular pressure gradient
TTE	Transthoracic Echocardiography
Vmax	Peak Aortic Jet Velocity
VTI	velocity-time integral

## References

[1] Dweck M.R., Chin C., Newby D.E. (2013). Small valve area with low-gradient aortic stenosis: Beware the hard hearted. J. Am. Coll. Cardiol..

[2] Baumgartner H., Hung J., Bermejo J., Chambers J.B., Edvardsen T., Goldstein S., Lancellotti P., Lefevre M., Miller F., Otto C.M. (2017). Recommendations on the Echocardiographic Assessment of Aortic Valve Stenosis: A Focused Update from the European Association of Cardiovascular Imaging and the American Society of Echocardiography. J. Am. Soc. Echocardiogr..

[3] Hachicha Z., Dumesnil J.G., Bogaty P., Pibarot P. (2007). Paradoxical Low-Flow, Low-Gradient Severe Aortic Stenosis Despite Preserved Ejection Fraction Is Associated with Higher Afterload and Reduced Survival. Circulation.

[4] Minners J., Allgeier M., Gohlke-Baerwolf C., Kienzle R.-P., Neumann F.-J., Jander N. (2007). Inconsistencies of echocardiographic criteria for the grading of aortic valve stenosis. Eur. Heart J..

[5] Clavel M.A., Messika-Zeitoun D., Pibarot P., Aggarwal S.R., Malouf J., Araoz P.A., Michelena H.I., Cueff C., Larose E., Capoulade R. (2013). The complex nature of discordant severe calcified aortic valve disease grading: New insights from combined Doppler echocardiographic and computed tomographic study. J. Am. Coll. Cardiol..

[6] Baumgartner H., Falk V., Bax J.J., De Bonis M., Hamm C., Holm P.J., Iung B., Lancellotti P., Lansac E., Munoz D.R. (2017). 2017 ESC/EACTS Guidelines for the management of valvular heart disease. Eur. Heart J..

[7] Otto C.M., Nishimura R.A., Bonow R.O., Carabello B.A., Erwin J.P., Gentile F., Jneid H., Krieger E.V., Mack M., McLeod C. (2021). 2020 ACC/AHA Guideline for the Management of Patients with Valvular Heart Disease: Executive Summary: A Report of the American College of Cardiology/American Heart Association Joint Committee on Clinical Practice Guidelines. Circulation.

[8] Hagendorff A., Knebel F., Helfen A., Knierim J., Sinning C., Stöbe S., Fehske W., Ewen S. (2019). Expert consensus document on the assessment of the severity of aortic valve stenosis by echocardiography to provide diagnostic conclusiveness by standardized verifiable documentation. Clin. Res. Cardiol..

[9] Nguyen T.-Q., Hansen K.L., Bechsgaard T., Lönn L., Jensen J.A., Nielsen M.B. (2018). Non-Invasive Assessment of Intravascular Pressure Gradients: A Review of Current and Proposed Novel Methods. Diagnostics.

[10] Dumesnil J.G., Pibarot P., Akins C. (2008). New approaches to quantifying aortic stenosis severity. Curr. Cardiol. Rep..

[11] Baumgartner H., Stefenelli T., Niederberger J., Schima H., Maurer G. (1999). “Overestimation” of catheter gradients by doppler ultrasound in patients with aortic stenosis: A predictable manifestation of pressure recovery. J. Am. Coll. Cardiol..

[12] Benfari G., Nistri S., Cerrito L.F., Maritan L., Tafciu E., Setti M., Bursi F., Tadiello E., De Manna N.D., Rossi A. (2020). Usefulness of the Right Parasternal Echocardiographic View to Improve the Hemodynamic Assessment After Valve Replacement for Aortic Stenosis. Am. J. Cardiol..

[13] Kumar G., Saikrishnan N., Sawaya F.J., Lerakis S., Yoganathan A.P. (2014). Response to letter regarding article, “accurate assessment of aortic stenosis: A review of diagnostic modalities and hemodynamics”. Circulation.

[14] Pibarot P., Clavel M.-A. (2015). Left Ventricular Outflow Tract Geometry and Dynamics in Aortic Stenosis: Implications for the Echocardiographic Assessment of Aortic Valve Area. J. Am. Soc. Echocardiogr..

[15] Leye M., Brochet E., Lepage L., Cueff C., Boutron I., Detaint D., Hyafil F., Iung B., Vahanian A., Messika-Zeitoun D. (2009). Size-Adjusted Left Ventricular Outflow Tract Diameter Reference Values: A Safeguard for the Evaluation of the Severity of Aortic Stenosis. J. Am. Soc. Echocardiogr..

[16] Vulesevic B., Kubota N., Burwash I.G., Cimadevilla C., Tubiana S., Duval X., Nguyen V., Arangalage D., Chan K.L., E Mulvihill E. (2020). Size-adjusted aortic valve area: Refining the definition of severe aortic stenosis. Eur. Heart J. Cardiovasc. Imaging.

[17] Park S.-J., Dweck M.R. (2019). Multimodality Imaging for the Assessment of Severe Aortic Stenosis. J. Cardiovasc. Imaging.

[18] Hahn R.T., Little S.H., Monaghan M.J., Kodali S.K., Williams M., Leon M.B., Gillam L.D. (2015). Recommendations for comprehensive intraprocedural echocardiographic imaging during TAVI. JACC Cardiovasc. Imaging.

[19] Lancellotti P., Pellikka P.A., Budts W., Chaudhry F.A., Donal E., Dulgheru R., Edvardsen T., Garbi M., Ha J.-W., Kane G.C. (2016). The clinical use of stress echocardiography in non-ischaemic heart disease: Recommendations from the European Association of Cardiovascular Imaging and the American Society of Echocardiography. Eur. Heart J. Cardiovasc. Imaging.

[20] Lancellotti P., Magne J., Piérard L.A. (2013). The role of stress testing in evaluation of asymptomatic patients with aortic stenosis. Curr. Opin. Cardiol..

[21] Huded C.P., Masri A., Kusunose K., Goodman A.L., Grimm R.A., Gillinov A.M., Johnston D.R., Rodriguez L.L., Popovic Z.B., Svensson L.G. (2018). Outcomes in Asymptomatic Severe Aortic Stenosis with Preserved Ejection Fraction Undergoing Rest and Treadmill Stress Echocardiography. J. Am. Heart Assoc..

[22] Postolache A., Nguyen M.L., Julien T., Sperlongano S., Chitroceanu A.M., Dulgheru R., Lancellotti P. (2020). Exercise echocardiography in aortic stenosis with preserved ejection fraction. Anatol. J. Cardiol..

[23] Maréchaux S., Hachicha Z., Bellouin A., Dumesnil J.G., Meimoun P., Pasquet A., Bergeron S., Arsenault M., Le Tourneau T., Ennezat P.V. (2010). Usefulness of exercise-stress echocardiography for risk stratification of true asymptomatic patients with aortic valve stenosis. Eur. Heart J..

[24] Lancellotti P., Donal E., Magne J., O’Connor K., Moonen M.L., Cosyns B., Pierard L.A. (2010). Impact of global left ventricular afterload on left ventricular function in asymptomatic severe aortic stenosis: A two-dimensional speckle-tracking study. Eur. J. Echocardiogr..

[25] Lancellotti P., Donal E., Magne J., Moonen M., O’Connor K., Daubert J.-C., Pierard L.A. (2010). Risk stratification in asymptomatic moderate to severe aortic stenosis: The importance of the valvular, arterial and ventricular interplay. Heart.

[26] Zito C., Salvia J., Cusmà-Piccione M., Antonini-Canterin F., Lentini S., Oreto G., Di Bella G., Montericcio V., Carerj S. (2011). Prognostic Significance of Valvuloarterial Impedance and Left Ventricular Longitudinal Function in Asymptomatic Severe Aortic Stenosis Involving Three-Cuspid Valves. Am. J. Cardiol..

[27] Reid A., Blanke P., Bax J.J., Leipsic J. (2020). Multimodality imaging in valvular heart disease: How to use state-of-the-art technology in daily practice. Eur. Heart J..

[28] Galli E., Fournet M., Chabanne C., Leguerrier A., Mabo P., Lelong B., Flecher E., Donal E. (2015). Prognostic value of left atrial reservoir function in patients with severe aortic stenosis: A 2D speckle-tracking echocardiographic study. Eur. Heart J. Cardiovasc. Imaging.

[29] Généreux P., Pibarot P., Redfors B., Mack M.J., Makkar R.R., A Jaber W., Svensson L.G., Kapadia S., Tuzcu E.M., Thourani V.H. (2017). Staging classification of aortic stenosis based on the extent of cardiac damage. Eur. Heart J..

[30] Tastet L., Tribouilloy C., Maréchaux S., Vollema E.M., Delgado V., Salaun E., Shen M., Capoulade R., Clavel M.-A., Arsenault M. (2019). Staging Cardiac Damage in Patients with Asymptomatic Aortic Valve Stenosis. J. Am. Coll. Cardiol..

[31] Rosa V., Ribeiro H.B., Sampaio R.O., Morais T.C., Rosa M.E., Pires L.J., Vieira M.L., Jr W.M., Rochitte C.E., de Santis A.S. (2019). Myocardial Fibrosis in Classical Low-Flow, Low-Gradient Aortic Stenosis. Circ. Cardiovasc. Imaging.

[32] Vamvakidou A., Jin W., Danylenko O., Pradhan J., Li W., West C., Khattar R., Senior R. (2019). Impact of Pre-Intervention Transaortic Flow Rate Versus Stroke Volume Index on Mortality Across the Hemodynamic Spectrum of Severe Aortic Stenosis: Implications for a New Hemodynamic Classification of Aortic Stenosis. JACC Cardiovasc. Imaging.

[33] Clavel M.-A., Magne J., Pibarot P. (2016). Low-gradient aortic stenosis. Eur. Heart J..

[34] Magne J., Donal E., Davin L., O’Connor K., Szymanski C., Piérard L.A., Lancellotti P. (2012). 194 Clinical outcome in asymptomatic severe aortic stenosis. Insights from the new proposed aortic stenosis grading classification. J. Am. Coll. Cardiol..

[35] Côté N., Simard L., Zenses A., Tastet L., Shen M., Clisson M., Clavel M. (2017). Impact of Vascular Hemodynamics on Aortic Stenosis Evaluation: New Insights into the Pathophysiology of Normal Flow—Small Aortic Valve Area—Low Gradient Pattern. J. Am. Heart Assoc..

[36] Blais C., Burwash I.G., Mundigler G., Dumesnil J.G., Loho N., Rader F., Baumgartner H., Beanlands R.S., Chayer B., Kadem L. (2006). Projected Valve Area at Normal Flow Rate Improves the Assessment of Stenosis Severity in Patients with Low-Flow, Low-Gradient Aortic Stenosis. Circulation.

[37] Clavel M.-A., Burwash I.G., Mundigler G., Dumesnil J.G., Baumgartner H., Bergler-Klein J., Sénéchal M., Mathieu P., Couture C., Beanlands R. (2010). Validation of Conventional and Simplified Methods to Calculate Projected Valve Area at Normal Flow Rate in Patients with Low Flow, Low Gradient Aortic Stenosis: The Multicenter TOPAS (True or Pseudo Severe Aortic Stenosis) Study. J. Am. Soc. Echocardiogr..

[38] Annabi M.-S., Touboul E., Dahou A., Burwash I.G., Bergler-Klein J., Enriquez-Sarano M., Orwat S., Baumgartner H., Mascherbauer J., Mundigler G. (2018). Dobutamine Stress Echocardiography for Management of Low-Flow, Low-Gradient Aortic Stenosis. J. Am. Coll. Cardiol..

[39] Simard L., Cote N., Dagenais F., Mathieu P., Couture C., Trahan S., Bosse Y., Mohammadi S., Page S., Joubert P. (2017). Sex-Related Discordance Between Aortic Valve Calcification and Hemodynamic Severity of Aortic Stenosis: Is Valvular Fibrosis the Explanation?. Circ. Res..

[40] Clavel M.A., Pibarot P., Messika-Zeitoun D., Capoulade R., Malouf J., Aggarval S., Araoz P.A., Michelena H.I., Cueff C., Larose E. (2014). Impact of aortic valve calcification, as measured by MDCT, on survival in patients with aortic stenosis: Results of an international registry study. J. Am. Coll. Cardiol..

[41] Pawade T., Clavel M.-A., Tribouilloy C., Dreyfus J., Mathieu T., Tastet L., Renard C., Gun M., Jenkins W.S.A., Macron L. (2018). Computed Tomography Aortic Valve Calcium Scoring in Patients with Aortic Stenosis. Circ. Cardiovasc. Imaging.

[42] Shen M., Tastet L., Capoulade R., Larose E., Bedard E., Arsenault M., Chetaille P., Dumesnil J.G., Mathieu P., Clavel M.A. (2016). Effect of age and aortic valve anatomy on calcification and haemodynamic severity of aortic stenosis. Heart.

[43] Woldendorp K., Bannon P.G., Grieve S.M. (2020). Evaluation of aortic stenosis using cardiovascular magnetic resonance: A systematic review & meta-analysis. J. Cardiovasc. Magn. Reson..

[44] Weininger M., Sagmeister F., Herrmann S., Lange V., Schoepf U.J., Beissert M., Voelker W., Koestler H., Hahn D., Weidemann F. (2011). Hemodynamic assessment of severe aortic stenosis: MRI evaluation of dynamic changes of vena contracta. Investig. Radiol..

[45] Azevedo C.F., Nigri M., Higuchi M.D.L., Pomerantzeff P.M., Spina G.S., Sampaio R.O., Tarasoutchi F., Grinberg M., Rochitte C.E. (2010). Prognostic Significance of Myocardial Fibrosis Quantification by Histopathology and Magnetic Resonance Imaging in Patients with Severe Aortic Valve Disease. J. Am. Coll. Cardiol..

[46] Magne J., Cosyns B., Popescu B.A., Carstensen H.G., Dahl J., Desai M.Y., Kearney L., Lancellotti P., Marwick T.H., Sato K. (2019). Distribution and Prognostic Significance of Left Ventricular Global Longitudinal Strain in Asymptomatic Significant Aortic Stenosis: An Individual Participant Data Meta-Analysis. JACC Cardiovasc. Imaging.

[47] Barone-Rochette G., Piérard S., Ravenstein C.D.M.D., Seldrum S., Melchior J., Maes F., Pouleur A.-C., Vancraeynest D., Pasquet A., Vanoverschelde J.-L. (2014). Prognostic Significance of LGE by CMR in Aortic Stenosis Patients Undergoing Valve Replacement. J. Am. Coll. Cardiol..

[48] Flett A.S., Hayward M.P., Ashworth M.T., Hansen M.S., Taylor A.M., Elliott P.M., McGregor C., Moon J.C. (2010). Equilibrium contrast cardiovascular magnetic resonance for the measurement of diffuse myocardial fibrosis: Preliminary validation in humans. Circulation.

[49] Lancellotti P., Vannan M.A. (2020). Timing of Intervention in Aortic Stenosis. N. Engl. J. Med..

[50] Bohbot Y., Renard C., Manrique A., Levy F., Maréchaux S., Gerber B.L., Tribouilloy C. (2020). Usefulness of Cardiac Magnetic Resonance Imaging in Aortic Stenosis. Circ. Cardiovasc. Imaging.

[51] Hwang J.-W., Kim S.M., Park S.-J., Cho E.J., Kim E.K., Chang S.-A., Lee S.-C., Choe Y.H., Park S.W. (2017). Assessment of reverse remodeling predicted by myocardial deformation on tissue tracking in patients with severe aortic stenosis: A cardiovascular magnetic resonance imaging study. J. Cardiovasc. Magn. Reson..

[52] Dweck M.R., Khaw H.J., Sng G.K., Luo E.L., Baird A., Williams M.C., Makiello P., Mirsadraee S., Joshi N.V., van Beek E.J.R. (2013). Aortic stenosis, atherosclerosis, and skeletal bone: Is there a common link with calcification and inflammation?. Eur. Heart J..

[53] Dweck M.R., Jones C., Joshi N.V., Fletcher A.M., Richardson H., White A., Marsden M., Pessotto R., Clark J.C., Wallace W.A. (2012). Assessment of Valvular Calcification and Inflammation by Positron Emission Tomography in Patients with Aortic Stenosis. Circulation.

[54] Dweck M., Jenkins W.S., Vesey A.T., Pringle M.A., Chin C., Malley T.S., Cowie W.J., Tsampasian V., Richardson H., Fletcher A. (2014). 18F-Sodium Fluoride Uptake Is a Marker of Active Calcification and Disease Progression in Patients with Aortic Stenosis. Circ. Cardiovasc. Imaging.

[55] Lindman B.R., Dweck M.R., Lancellotti P., Genereux P., Pierard L.A., O’Gara P.T., Bonow R.O. (2020). Management of Asymptomatic Severe Aortic Stenosis: Evolving Concepts in Timing of Valve Replacement. JACC Cardiovasc. Imaging.

[56] Mack M.J., Brennan J.M., Brindis R., Carroll J., Edwards F., Grover F., Shahian D., Tuzcu E.M., Peterson E.D., Rumsfeld J.S. (2013). Outcomes Following Transcatheter Aortic Valve Replacement in the United States. JAMA.

[57] Rapezzi C., Giannini F., Campo G. (2021). Aortic stenosis, transcatheter aortic valve replacement and transthyretin cardiac amyloidosis: Are we progressively unraveling the tangle?. Eur. J. Heart Fail..

[58] Nitsche C., Scully P.R., Patel K.P., Kammerlander A.A., Koschutnik M., Dona C., Wollenweber T., Ahmed N., Thornton G.D., Kelion A.D. (2020). Prevalence and Outcomes of Concomitant Aortic Stenosis and Cardiac Amyloidosis. J. Am. Coll. Cardiol..

[59] Ternacle J. (2020). PPaCM. Aortic Stenosis and Cardiac Amyloidosis. JACC Case Rep..

[60] Peix A., Padron K. (2021). Imagine, believe, and achieve. J. Nucl. Cardiol..

[61] Dorbala S., Ando Y., Bokhari S., Dispenzieri A., Falk R.H., Ferrari V.A., Fontana M., Gheysens O., Gillmore J.D., Glaudemans A.W.J.M. (2021). ASNC/AHA/ASE/EANM/HFSA/ISA/SCMR/SNMMI Expert Consensus Recommendations for Multimodality Imaging in Cardiac Amyloidosis: Part 2 of 2—Diagnostic Criteria and Appropriate Utilization. Circ. Cardiovasc. Imaging.

[62] Scully P.R., Patel K.P., A Treibel T., Thornton G.D., Hughes R.K., Chadalavada S., Katsoulis M., Hartman N., Fontana M., Pugliese F. (2020). Prevalence and outcome of dual aortic stenosis and cardiac amyloid pathology in patients referred for transcatheter aortic valve implantation. Eur. Heart J..

